# Are two internal thoracic grafts better than one in patients with chronic obstructive lung disease? Analysis of 387 cases between 1996-2011

**DOI:** 10.1371/journal.pone.0201227

**Published:** 2018-08-13

**Authors:** Pevni Dmitry, Aizer Zahi, Mohr Rephael, Nesher Nahum, Kremer Amir, Paz Yosef, Taih Nadav, Ben-Gal Yanai

**Affiliations:** 1 Department of Cardiothoracic Surgery Tel Aviv Sourasky Medical Center, Tel Aviv University, Tel Aviv, Israel; 2 Sackler Faculty of Medicine, Tel Aviv University, Tel Aviv, Israel; Universita degli Studi di Bologna, ITALY

## Abstract

**Objectives:**

Bilateral internal thoracic artery (ITA) grafting is associated with improved survival. However, potential survival benefit of using two ITA`s in patients with chronic lung disease (CLD) is questionable due to their increased risk of sternal wound infection (SWI) compared to operations incorporating single ITA (SITA). The purpose of this study is to compare early and long-term outcome of bilateral internal thoracic artery (BITA) grafting to that of grafting with single internal thoracic grafts and vein grafts or radial artery (SITA) in CLD patients with multi-vessels coronary disease.

**Methods:**

One hundred and forty eight CLD patients who underwent BITA between 1996 and 2011 were compared with 239 who underwent SITA at the same period.

**Results:**

SITA patients were more often female, more likely to have insulin treated diabetes (DM), DM with end organ damage, neurologic dysfunction and unstable angina.

Despite of the difference in preoperative characteristics, early mortality (5.4% vs. 5.4%, in the SITA and BITA respectively, p = 0 < .999) and occurrences of SWI (6.3% vs 9.5%, p = 0.320) and strokes (3.8% vs 5.4%,p = 0.611) were not significantly different between groups. BITA patients did not have better Kaplan-Meier 10 year survival (52.8% vs. 42.6%, p = 0.088) and after matching, BITA and SITA had similar adjusted survival (HR 0.983[95%CI 0.755–1.280] p = 0.901) (cox model).

**Conclusion:**

Our study results suggest that in patients with CLD, the choice of BITA grafting technique did not provide survival benefit compared to SITA with other conduits.

## Introduction

Internal Thoracic Artery (ITA) grafts have better long-term patency than Saphenous Vein Grafts (SVG's) and this better patency is believed to be responsible for the improved long term survival, when used as bypass to the left anterior descending artery [1, 2, 3]. The use of two ITA's was shown to further improve patients undergoing myocardial revascularization [4, 5, 6]. Besides the improved survival compared to single ITA (SITA) and saphenous vein grafting, bilateral ITA (BITA) patients, reportedly had better event-free survival and reduced occurrence of re-interventions [4, 5]. Extensive arterial grafting with BITAs in these reports had been used preferentially in a selected group of young male non-obese non-diabetic patients [4, 5, 6]. Patients were preselected for BITA grafting according to their life expectancy and only a few patients older than 70 years and very few with co-morbidities such as Chronic Lung Disease (CLD) were offered the option of BITA grafting.

Despite the improved long-term outcome of BITA grafting, in the general population of patients undergoing coronary artery bypass grafting (CABG), surgeons are reluctant to use this grafting technique in the subset of patients with CLD. The main reasons for this are the questionable survival benefit in light of their reported shorter life expectancy [7] and increased risk of perioperative mortality [8] and morbidity [8, 9]. The increased postoperative respiratory effort in patients with CLD[10–12] together with the more extensive revascularization associated with harvesting two ITA’s [13] causes increased risk of sternal wound infection (SWI). Despite of the above, during the study period (1996–2011) CLD was not a definite contraindication for employing the BITA grafting in Tel Aviv Sourasky Medical Center and BITA grafting was performed in 148 CLD patients.

The purpose of this report is to compare, early and long-term outcomes of SITA and BITA grafting in CLD patients with multivessel disease.

## Patients and methods

This retrospective review of the medical records for obtaining follow-up information was approved by the Institutional Review Board of the Tel Aviv Medical Center (informed consent was waived). Follow-up information, was completed (98.2%) by means of the Israeli National Registry database.

Between 1996 and 2011, 387 patients with multi-vessel coronary artery disease (CAD) and chronic lung disease (CLD) [14] underwent myocardial revascularization. They constituted 7.2% of all isolated CABG procedures for multi-vessel disease (5302 operations) performed in our institute during this time period. Unlike the practice described in previous reports [4–6], Left sided (LAD and Circumflex) arterial grafting with BITA was the preferred revascularization method in our center during the entire study period and patients were not utterly rejected for BITA grafting according to their life expectancy or co-morbidities.

There was however, a tendency among our surgeons not to use BITA grafts in patients with increased risk for sternal wound complications (i.e., elderly patients, patients with CLD or females with diabetes and/or obesity) [15–18], thus only 38.2%(148 patients) of the CLD cases included in this report were treated with BITA`s. The 148 CLD patients that underwent BITA grafting were compared with 239 multi-vessel CLD patients who underwent CABG with SITA and other conduits such as SVG, RA and rGEA.

### Surgical techniques

Operations were performed using standard cardiopulmonary bypass (CPB) or off-pump coronary artery bypass (OPCAB). Myocardial preservation during CPB involved intermittent blood cardioplegia (30–32°C). ITAs were harvested as skeletonized vessels [16]. In most cases, BITA were used to graft the left coronary system. Two graft arrangements were implemented: in-situ BITA grafting, with an ante-aortic crossover right ITA (RITA) to the LAD and left ITA (LITA) to the circumflex marginals and composite T-grafting with a free RITA attached proximally end-to-side on the LITA. [16, 19]

The choice of procedure type, BITA or SITA, was made mainly according to the surgeon's preference. The choice of BITA configuration was determined by previously detailed technical considerations [15, 16, 18]. A composite T-graft with the right ITA was mostly used when the target coronary vessel stenosis was >80% [16, 19, 20]. In addition our strategy was to use RITA (distal end of composite T grafts), right gastro-epiploic arteries and RAs as grafts to the RCA branches only in the presence of a significant RCA stenosis (i.e., >80%) [19, 20]. When the RCA stenosis was less than 80% we selected SVGs as the conduit for RCA revascularization. To decrease the risk of spasm of the arterial grafts, all patients were treated with a high-dose intravenous infusion of Isosorbide dinitrate (Isoket, 4–20 mg/h) during the first 48 postoperative hours. Calcium-channel blockers (Diltiazem, 90–180 mg/d, orally) were starting from the second postoperative day and continuing for at least three subsequent months to patients whose surgery included a right gastro-epiploic artery or an RA [15,16,18]. All patients were permanently treated by aspirin 100 mg daily and by statins.

### Definitions and data collection

Patient data were analyzed according to EuroSCORE clinical data standards [14]. Chronc Lung Disease (CLD) was defined as long–term use of bronchodilators or5 steroids for lung disease. Diabetes was classified as non-insulin treated diabetes mellitus (NIDDM) and as insulin treated (IDDM). A peri-operative myocardial infarction (MI) was defined as the postoperative appearance of new Q waves or ST segment elevation of more than 2 mm on an electrocardiograph, accompanied by a creatine phosphokinase-myocardial band greater than 50 mU/mL, with or without a regional wall motion abnormality [21–24]. A cerebrovascular accident was defined as a new permanent neurological deficit and computed tomographic evidence of cerebral infarction. Deep sternal wound infection (SWI) in this setting was defined as the presence of deep infection in combination with late dehiscence requiring sternectomy. Early mortality was defined as mortality during the first thirty days after surgery or mortality within the same hospitalization. Our definition of "emergency operation" was based on the EuroSCORE and it includes patients operated on within 24 hours of cardiac catheterization [19,20] or those with ongoing angina, acute evolving MI, pulmonary edema or in cardiogenic shock[15,16,18].

### Statistical analysis

#### a. Complete cohort early outcome

All data were summarized and displayed as mean (±standard deviation) if normally distributed or median and range if non-normally distributed, and as the number (percentage) of patients in each group for categorical variables. Categorical variables were compared by using χ^2^ or Fisher’s exact tests, as appropriate. Continuous variables were compared using the independent sample t test or Mann–Whitney test.

#### b. Complete cohort late outcome

Follow-up which was 98.2% complete was obtained using the Israeli National Registry database. Median follow-up was 16.29 (Range 0–21.26) years. Log-rank test and Kaplan-Meier curves were used to compare survival among groups. Reverse censoring method was used to describe the length of follow-up period. The univariate and multivariate Cox regression was used to identify predictors of decreased survival. The type of conduit used (BITA or SITA) was forced into the regression model in the first block. Age and gender were forced into the regression model in the second block. The third block included potential predictors using forward stepwise selection method. Preoperative characteristics that were considered as potential predictors in the regression were: NIDDM, IDDM, Diabetes with End Organ Damage(DM+EOD) unstable angina(UAP), critical preoperative state, emergency operation, preoperative use of intra aortic balloon (IABP), neurologic dysfunction(ND), old MI, acute MI, chronic renal failure(CRF), repeat operation, peripheral-vascular disease (PVD), cerebro-vascular disease (CVD), congestive heart failure (CHF), preoperative per-cutaneous intervention (PCI), left main disease, the number of diseased vessels and, left ventricular EF. The next block was also performed using forward stepwise selection method and included intra-operative variables such as, other conduits used (SVG, RA, rGEA,), sequential grafting, the number of grafts constructed and operations performed without extracorporeal circulations (OPCAB). In the last block, we added the operative era (1996–2000 vs.2001-2011) which was forced into the regression.

#### C. Matched pairs outcome CLD patients only (BITA and SITA patients)

In order to control for baseline differences in BITA and SITA patients we performed propensity score matching [25]. Logistic regression was used to calculate the propensity score as the probability of patients to undergo BITA grafting. Age, sex, NIDDM, IDDM DM+ EOD, CHF,CRF, old MI, acute MI, unstable angina, EF ≥ 30%,IABP, critical preoperative state, emergency, repeat operation, PVD, CVD, ND, number of vessels ≥ 3, LM and PCI were included in the logistic regression. An absolute difference up to 5% in the propensity score was considered as acceptable for matching Paired-sample T-test, Wilcoxon test and Mcnamare test were used to compare the matched groups. Univariate and Multivariate Stratified–Cox regression were used to evaluate the association between grafting technique and mortality during the follow-up period. The multivariable analysis included grafting technique and intra-operative variables. Backward method was used for variable selection (WALD-test as criteria, p>0.1 for removal).

#### D. Matched pairs outcome (CLD with non-CLD patients

In order to show the effect of conduit used in CLD patients on early and late outcomes we described the effect in two cohorts of patients with the same age, and sex distributions as well as similar co-morbidities, one with CLD and one without CLD. In order to get two cohort of patients with similar background characteristics we matched the CLD cohort with non CLD cohort operated in the same period according to age±2years, gender, Non-Insulin treated diabetes (NIDDM), EuroSCORE±2%, congestive heart failure (CHF), peripheral vascular disease (PVD), and old MI.

The McNemar's test and paired sample t test were used to compare discrete and continuous variables that were not included in the matching criteria. A new parameter with 4 categories was defined using CLD status and conduit type (Non CLD–SITA, Non CLD–BITA, CLD–SITA, CLD–BITA). Univariate and multivariate stratified Cox regressions were used to compare long term outcomes among the four categories created. Variable selection for inclusion in the multivariate analyses was performed as described previously.

All tests were two tailed and p<0.05 was considered significant. Statistical analysis was performed with SPSS software (IBM SPSS Statistics, IBM corp, 2013, Version 22, Armink, NY, USA).

## Results

### Preoperative CLD patient's characteristics

There were significant differences in preoperative characteristics between patients treated with SITA and patients treated with BITA (Table 1). There were more females as well as a higher rate of Congestive heart failure [CHF], unstable angina, insulin-treated diabetes, diabetes with end organ damage (DM+EOD) and neurologic dysfunction (ND) in the SITA group. The mean EuroSCORE of the SITA group was significantly higher than that of the BITA group (9.04±4.32 vs. 7.69±3.11, *p* < 0.001) (EuroSCORE in Table 1).

**Table 1:** Patients characteristics before matching (n = 387).

Factor	Total (n = 387)		BITA (n = 148)		SITA (n = 239)		P
**Age≤65**	124	32.0%	53	35.8%	71	29.7%	0.155
Age66-75	173	44.7%	68	45.9	105	43.9%	0.155
Age76+	90	23.3%	27	18.2%	63	26.4%	0.155
**Female gender**	94	24.3%	27	18.2%	67	28.0%	0.029
**NIDDM**	125	32.3%	45	30.4%	80	33.5%	0.323
**IDDM**	24	6.2%	4	2.7%	20	8.4%	0.025
**DM+EOD**	51	13.2%	11	7.4%	40	16.7%	0.009
CHF	114	29.5%	50	33.8%	64	26.8%	0.142
CRF(Cr ≥1.8)	54	14.0%	15	10.1%	39	16.3%	0.088
**Peripheral vascular disease**	115	29.7%	50	33.8%	65	27.2%	0.168
**Cerebral vascular disease**	73	18.9%	23	15.5%	50	20.9%	0.189
**ND**	30	7.8%	6	4.1%	24	10.0%	0.032
**EF ≤30%**	45	11.6%	14	9.5%	31	13.0%	0.295
**Unstable angina pectoris**	297	63.8%	84	56.8%	163	68.2%	0.023
**Acute MI ≤1 week**	88	22.7%	39	26.4%	49	20.5%	0.182
**Recent MI ≤3 months**	134	34.6%	52	35.1%	82	34.3%	0.868
**Old MI>1 week**	184	47.5%	63	42.6%	121	50.6%	0.123
**3-Vessel disease**	274	70.8%	107	72.3%	167	69.9%	0.610
**Left main disease**	108	27.9%	49	33.1%	59	27.4%	0.073
**Preoperative IABP**	43	11.1%	14	9.5%	24	11.5%	0.325
Critical preoperative state*	38	9.8%	18	12.2%	20	8.4%	0.223
**Emergency operation**	81	21.9%	27	18.2%	54	22.6%	0.307
**S/P PCI**	65	16.8%	27	18.2%	38	15.9%	0.549
**Repeat operation**	11	2.8%	4	2.7%	7	2.9%	0.582
**Euro SCORE (mean±SD)**	8.51±4.09		7.69±3.81		9.04±4.32		<0.001

BITA, bilateral internal thoracic artery; SITA, single internal thoracic artery; NIDDM, non-insulin-dependent diabetes mellitus; IDDM, insulin-dependent diabetes mellitus; MI, myocardial infarction; EF, ejection fraction; IABP, intra-aortic balloon pump; CRF, chronic renal failure; EOD, End Organ Damage; ND, neurologic dysfunction; CHF, congestive heart failure; PCI, percutaneous intervention;

*EuroSCORE definition[14].

### Perioperative data and early results

More patients in the BITA group underwent three or more bypass grafts, however more patients in the SITA group underwent revascularization of the right coronary system (RCA revascularization) and sequential grafting (Table 2). Operative mortality (5.4% vs. 5.4%, SITA and BITA respectively, *p* = <0.999), all cause early mortality demonstrated in Table 3. Occurrence of sternal wound infection (6.3% vs. 9.5%, *p* = 0.320) and strokes (3.8% vs. 5.4%, *p* = 0.611) were similar among the two groups (Table 4).

**Table 2:** Surgical data.

	Bilateral ITA n = 148	Single ITA n = 239	p
Sequential grafting	74 (50.0%)	104 (43.5%)	0.213
Use of SVG	46 (31.1%)	159 (66.5%)	<0.001
Use of RA	6 (4.1%)	73 (30.5%)	<0.001
Use of r GEA	21 (14.2%)	19 (7.9%)	0.050
RCA revascularization	88 (59.5%)	179 (74.9%)	0.001
OPCAB	31 (20.9%)	69 (28.9%)	0.084
≥3 Bypass grafts	105 (70.9%)	152 (63.6%)	0.177

RCA, right coronary artery; SVG, saphenous vein graft; ITA, internal thoracic artery; OPCAB, Off Pump Coronary Bypass Grafting; RA, radial artery; rGEA, right gastroepiploic artery.

**Table 3:** All cause of early mortality.

Factor	Single ITA N = 13	Bilateral ITA N = 8
Cardiogenic shock	4 (30.7%)	2 (25.0%)
CVA	3 (23.1%)	2 (25.0%)
Sepsis	5 (47.2%)	3 (37.5%)
Sudden death	0	1 (12.5%)

ITA, internal thoracic artery; MI, myocardial infarction. CVA -Cerebrovascular accident

**Table 4:** Early mortality and morbidity.

Factor	Bilateral ITA n = 148	Single ITA n = 239	p
30-day mortality	13 (5.4%)	8 (5.4%)	1.000
Perioperative MI	7 (2.9%)	4 (2.7)	1.000
Postoperative stroke	9 (3.8%)	8 (5.4%)	0.611
Sternal wound infection	15 (6.3%)	14 (9.5%)	0.320

ITA, internal thoracic artery; MI, myocardial infarction.

### Long-term outcome

Follow-up was 98.2% complete. Median follow-up was 16.29(range 0–21.26) years.

**Fig 1:**
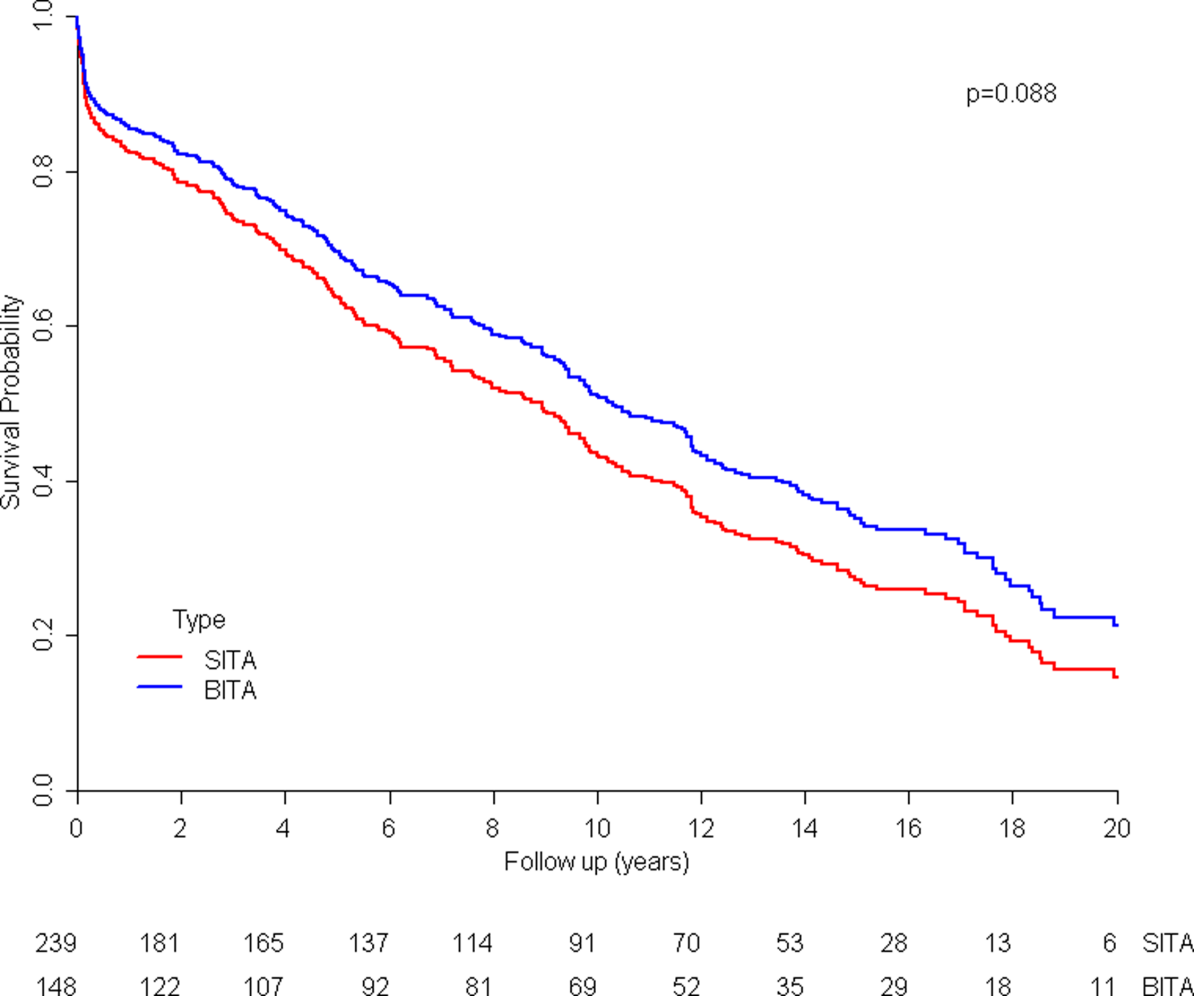
Kaplan-Meier curves comparing survival of patients before matching: BITA vs. SITA.

The 5, 10 and 15-years survival in the SITA group and BITA group were 63.5, 42.6, 27.3 and 69.7, 52.8, 34.6 respectively. Overall, there was no significant difference in the survival of the two groups *p = 0*.*088* (Fig 1). Similar results were obtained after adjustment, for potential confounders (Table 5) (Cox model).

**Table 5:** Independent predictors for overall (early +late) mortality (Cox).

FACTOR	PROBABILITY	p
Age Sex	HR 1.057, 95% CI 1.109–1.041 HR 1.129, 95% CI 0.850–1.501	0.000 0.402
IDDM	HR 2.635, 95% CI 1.642–4.227	0.000
DM+EOD EF ≤30% Emergency operation Year 2001–2011 Conduit BITA	HR 1.419, 95% CI 0.999–2.015 HR 1.791, 95% CI 1.239–2.590 HR 1.286, 95% CI 0.957–1.729 HR 0.967, 95% CI 0.735–1.272 HR 0.983, 95% CI 0.755–1.280	0.051 0.002 0.095 0.809 0.901

EF, ejection fraction; IDDM, insulin-dependent diabetes mellitus; DM+EOD Diabetes with End Organ Damage; Conduit BITA (Bilateral Internal Thoracic Artery)]

Advance age, IDDM and EF ≤30%, were predictors of increased operative mortality (Table 5).

Using Multivariate Stratified Cox regression BITA grafting was not associated with increased long-term mortality (HR = 1.04,95% CI 0.78–1.37,p = 0.798)

### Matched pairs results

There were significant differences in preoperative characteristics between patients treated with SITA and patients treated with BITA (Table 6). There were more females as well as, non insulin-treated diabetes, diabetes with end organ damage (DM+EOD), EF ≤30%, Recent and Old MI, Left main disease, Emergency, Critical preoperative state,and preoperative use of IABP in the SITA group. The mean EuroSCORE of the SITA group was significantly higher than that of the BITA group (8.53±4.38 vs. 7.61±3.84, *p* = 0.081) (EuroSCORE in Table 6).

**Table 6:** Patients characteristics after matching (n = 266).

Factor	Total (n = 266)		BITA (n = 133)		SITA (n = 133)		P
Mean age			68.27 ± 9.78		67.53 ± 9.29		0.533
Female gender	57	21.4%	26	19.5%	31	23.3%	0.568
NIDDM	87	32.7%	42	31.6%	45	33.8%	0.798
IDDM	9	3.4%	4	3.0%	5	3.8%	<0.999
DM+EOD	23	8.6%	10	7.5%	13	9.8%	0.668
CHF CRF(Cr ≥1.8)	83 31	31.2% 11.7%	41 15	30.8% 11.3%	42 16	31.6%12.0%	<0.999 <0.999
Peripheral vascular disease	81	30.5%	40	30.1%	41	30.8%	<0.999
Cerebral vascular disease	41	15.4%	20	15.0%	21	15.8%	<0.999
ND	11	4.1%	6	4.5%	5	3.8%	<0.999
EF ≤30%	28	10.5%	13	9.8%	15	11.3%	0.839
Unstable angina pectoris	158	59.4%	79	59.4%	78	58.4%	<0.999
Acute MI ≤1 week	61	22.9%	31	23.3%	30	22.6%	<0.999
Recent MI ≤3 months	90	33.8%	44	33.1%	46	34.6%	0.899
Old MI>1 week	119	44.7%	58	43.6%	61	45.9%	0.780
3-Vessel disease	188	70.7%	94	70.7%	94	70.7%	<0.999
Left main disease	84	31.6%	39	29.3%	45	33.8%	0.526
Preoperative IABP	29	10.9%	13	9.8%	16	12.0%	0.690
Critical preoperative state*	30	11.3%	14	10.5%	16	12%	0.839
Emergency operation	53	19.9%	24	18.0%	29	21.8%	0.500
S/P PCI	49	18.4%	24	18.0%	25	18.8%	<0.999
Repeat operation	7	2.6%	3	2.3%	4	3.0%	<0.999
Euro SCORE (mean±SD)			8.53±4.38		7.61±3.84		<0.081

BITA, bilateral internal thoracic artery; SITA, single internal thoracic artery; NIDDM, non-insulin-dependent diabetes mellitus; IDDM, insulin-dependent diabetes mellitus;; MI, myocardial infarction; EF, ejection fraction; IABP, intra-aortic balloon pump; CRF, chronic renal failure; EOD, End Organ Damage; ND, neurologic dysfunction; CHF, congestive heart failure; PCI, percutaneous intervention;

*EuroSCORE definition[14].

The 357 patients were also matched for CLD versus Non CLD. After matching CLD and Non CLD patients were not statistically different (p>0.05 for all variables evaluated). Non of the studied short and long term outcomes were significantly different between the four patients categories (non CLD–SITA, non CLD–BITA, CLD–SITA and CLD–BITA).

**Fig 2:**
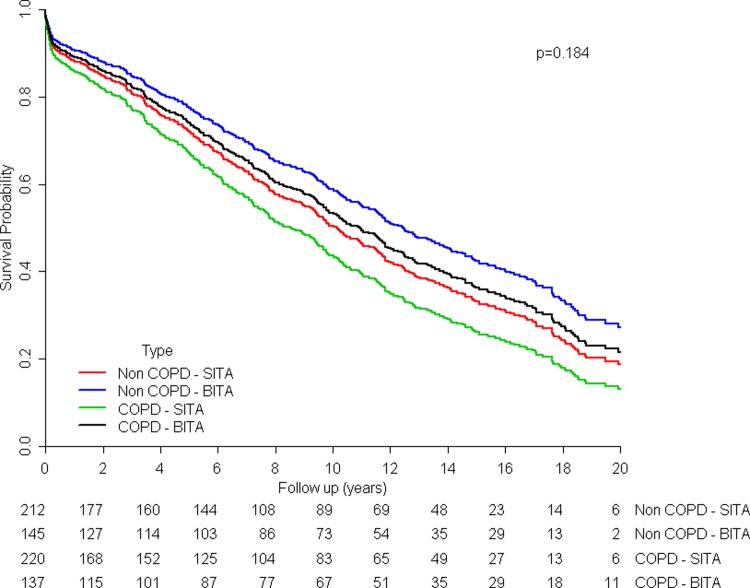
Kaplan-Meier survival curves of the groups after matching.

One hundred and thirty nine (65.6%) patients in the non CLD–SITA, 80 (55.2%) in the non CLD–BITA, 161 (73.2%) in the CLD–SITA and 91 (66.4%) in the CLD–BITA died during the follow up period (p = 0.184, Fig 2). After adjustment for potential confounders, the significance level almost remained the same (p = 0.114).

## Discussion

This retrospective analysis evaluated the early and long-term clinical outcome of CLD patients with multi-vessel disease who underwent CABG.

CLD patients who underwent BITA grafting were compared with CLD patients who underwent SITA grafting and other conduits, such as SVGs or RAs.

The main finding in this report is the similar long-term (9.98 ± 9.21 years) survival of BITA grafting in COPD patients, compared to SITA grafting. This observation contrasts with the similar long term survival of SITA and BITA patients of the No–COPD matched cases with similar preoperative characteristics generated from the whole cohort. This finding suggest that the decreased survival of the COPD patient is probably related to the COPD and not to other preoperative risk factors.

The use of ITA grafts is known to be associated with postoperative pulmonary problems [26]. COPD patients had increased tendency for postoperative atrial-fibrillation and slightly longer hospital stays compared to patients with normal spirometry. This may be related to the associated lung disease or to the use of short-acting bronchodilators [27, 28]. Approximately 25% of the severe COPD patients had increased occurrences of postoperative bronchitis episodes and pneumonia.

In a report by Manganas et al COPD patients were categorized according to lung disease severity and lung spirometry. :In this report, ITA was used in only 70% of the cases with severe and moderate lung disease(FEV1/FVC 0.7 or less.) compared to 86% in the Non-COPD control group [29].

Unlike other studies reporting results of CABG in patients with COPD, at least one ITA was used in all patients included in the current report. Routine left-sided arterial (LAD and Circumflex system) revascularization was the preferred method of revascularization in our center during the study period [15, 16]. Thus, most (3028 patients, 58.0%) of the primary CABG procedures for multi-vessel disease that were performed in our institution during the study period were skeletonized BITA grafting.

Several studies reported extensive arterial grafting with BITA as preferential treatment among selected groups, such as young male, non-obese, non-diabetic patients [4, 5, 6]. In those studies, patients were preselected for BITA grafting according to their life expectancy, and patients with co-morbidities such as COPD, or age above 70 years were rarely offered BITA grafting.

Selection of surgical approach for the current cohort was based on the surgeons' decisions. The main criterion considered for BITA was a reduced risk of deep sternal wound complications; high-risk patients (with COPD, and obese and diabetic women) were preferentially referred to the SITA group during the study period [17]. Therefore, unlike in previous report from our center[15, 16, 30, 31, 32], most of the patient included in the current reports (Un-matched COPD patients) were patients treated with SITA. Therefore, SITA patients were more often female, and more likely to have comorbidities such as insulin treated Diabetes(DM), DM with end organ damage, neurologic dysfunction and unstable angina. The mean Euro-score of the SITA group was significantly higher than that of the BITA group (9.05±4.32 vs. 7.69±3.11, *p* < 0.001).

Case control matching was used to account for differences between COPD and No-COPD patients in preoperative demographic and clinical characteristics. The risk profile of the matched groups was largely determined by the higher risk of the COPD group (EuroSCORE 7.58±3.44). This can explain the high occurrences of co-morbidities, emergency operations and the relatively unfavorable long term outcomes of patients in our study compared to those in other BITA series [4, 5, 6].

Despite the use of skeletonized ITAs [33], the rate of sternal wound infection was considerably high. This may be partially explained by the non-selective use of BITA and by the fact that precautions such as preoperative nasal swabs, nasal disinfection, strict postoperative glucose control and enhanced sternal wound stabilization were not yet employed during the early years of the study (1996–2000). Increased prevalence of CVD, PVD, NIDDM and the fact that a significant proportion of the BITA patients were operated before introduction of epi-aortic ultra-sound, preoperative CT and popularization of the aortic no-touch OPCAB technique may also explain the relatively high occurrence of strokes.

Occurrences of early post-operative complications (Operative mortality, SWI, perioperative MI, and postoperative stroke) did not differ significantly between the BITA and SITA groups. Moreover, the type of conduit that was employed was not a significant predictor of mortality, SWI, perioperative MI or stroke.

This similar outcome seem to justify the surgeon’s preference selection strategy for BITA or SITA grafting based on the risk for SWI. However, this study fails to justify that same strategy in terms of long-term survival which is the main goal of BITA revascularization.

The better long-term survival of BITA patients in the previous reports [4, 5, 6, 34, 35], unlike in our report, may stem from the fact that BITA patients in our study were at significantly higher risk and older (CLD and other co-morbidities)

### Limitations

1. The single center observational retrospective design of our study is a definite limitation.
2. The main cause of late mortality was not collected and therefore was not reported. Follow up clinical data was not available for all MACE (Major Adverse Events). Thus, end points such as late MI, cardiac mortality and re-interventions that were not collected prospectively, were not complete, and their occurrences could not be compared between groups. Therefore, MACE was not assessed in the analysis. Given the sample size of the matched groups, it is likely that event free survival may have revealed differences between groups.
3. Complete postoperative angiographic data and data on performance of postoperative PCI were not collected and therefore were not reported in this study.
4. Another limitation is the possible selection bias in the criteria used for the choice of the second conduit and the tendency of surgeons not to use BITA grafting in patients with increased risk for sternal wound complications (COPD, obese diabetic females, etc.). Therefore, patients treated with SITA were older and higher risk patients.

In conclusion, our study results suggest that in patients with CLD, the choice of BITA grafting technique did not provide survival benefit compare to SITA with other conduits.

## Supporting information

S1 Dataset Dataset for this study. (XLSX)
