# Correction: Are two internal thoracic grafts better than one in patients with chronic obstructive lung disease? Analysis of 387 cases between 1996-2011

**DOI:** 10.1371/journal.pone.0208046

**Published:** 2018-11-19

**Authors:** Dmitry Pevni, Zahi Aizer, Rephael Mohr, Nahum Nesher, Amir Kremer, Yosef Paz, Nadav Taih, Yanai Ben-Gal

There are multiple errors in the author byline. Please view the correct author order, affiliations, and citation here:

Dmitry Pevni^1,2^, Zahi Aizer^2^, Rephael Mohr^1,2^, Nahum Nesher^1,2^, Amir Kremer^1,2^, Yosef Paz^1,2^, Nadav Taih^1,2^, Yanai Ben-Gal^1,2^

1 Department of Cardiothoracic Surgery Tel Aviv Sourasky Medical Center, Tel Aviv University, Tel Aviv, Israel, 2 Sackler Faculty of Medicine, Tel Aviv University, Tel Aviv, Israel

Pevni D, Aizer Z, Mohr R, Nesher N, Kremer A, Paz Y, et al. (2018) Are two internal thoracic grafts better than one in patients with chronic obstructive lung disease? Analysis of 387 cases between 1996–2011. PLoS ONE 13(8): e0201227. https://doi.org/10.1371/journal.pone.0201227.
